# Characteristics and Epidemiology of Extended-Spectrum β-Lactamase-Producing Multidrug-Resistant *Klebsiella pneumoniae* From Red Kangaroo, China

**DOI:** 10.3389/fmicb.2020.560474

**Published:** 2020-10-14

**Authors:** Xue Wang, Qian Kang, Jianan Zhao, Zhihui Liu, Fang Ji, Junbao Li, Jianchun Yang, Chenglin Zhang, Ting Jia, Guoying Dong, Shelan Liu, Guocheng Hu, Jianhua Qin, Chengmin Wang

**Affiliations:** ^1^Guangdong Key Laboratory of Animal Conservation and Resource Utilization, Guangdong Public Laboratory of Wild Animal Conservation and Utilization, Institute of Zoology, Guangdong Academy of Science, Guangzhou, China; ^2^College of Veterinary Medicine, Agricultural University of Hebei, Baoding, China; ^3^Zhengzhou Zoo, Zhengzhou, China; ^4^Beijing Key Laboratory of Captive Wildlife Technologies, Beijing Zoo, Beijing, China; ^5^College of Global Change and Earth System Science, Beijing Normal University, Beijing, China; ^6^Department of Infectious Diseases, Zhejiang Provincial Centre for Disease Control and Prevention, Hangzhou, China; ^7^South China Institute of Environmental Sciences, Ministry of Ecology and Environment, Guangzhou, China

**Keywords:** multidrug-resistance, *Klebsiella pneumonia*, genomic characteristic, extended-spectrum β-lactamase-producing, public health risk5c

## Abstract

Due to its drug resistant nature, β-lactamase represents a serious challenge for public health. Extended-spectrum β-lactamase (ESBL) producing *Klebsiella pneumoniae* clones are increasingly reported worldwide. Little is known about the prevalence and biological characteristics of drug-resistant strains in zoos. During routine surveillance at the Zhengzhou Zoo of China, we found *Klebsiella pneumoniae* isolate in healthy Red Kangaroos (*Macropus Rufus*) with severe MDR. The *Klebsiella pneumoniae* were especially resistant to Cefuroxime Sodium (MIC, > 64 μg/mL), Ceftriaxone (MIC, >8 μg/mL) and Cefepime (MIC, >64 μg/mL), and belonged to ST290. Subsequently, whole genome sequencing (WGS) showed that the Chrome Chr-M297-1 harbored *bla*_DHA–3_, *bla*_SHV–1_, *bla*_CTX–M–14_, *fosA5*, *dfrA3*, *sul3*, etc., and pM297-1.1 [222,864 bp, IncFIB(K)], which carried nine antimicrobial genes including *bla*_CTX–M–14_, *bla*_TEM–191_, *aph(3″)-Ib*, *aph(6)-Id* and *qnrS1*, etc., and pM297-1.2 [225,763 bp, IncFII(K)] carried 22 antimicrobial genes including *bla*_TEM–1_, *bla*_CTX–M–3_, *aph(3′)-Ia*, *aac(3)-IIa*, *aac(6′)-Ib-cr*, *aadA16*, *qnrB2*, *qnrS1*, *qacEΔ1*, *mphA*, *sul1*, and *dfrA27*, etc. A traceability analysis then revealed that these two plasmids were highly similar to those recovered from human clinical samples in some southern cities in Sichuan Province, China (>99%), suggesting that these plasmids are spreading in China. Furthermore, two plasmids harboring conjugal transfer genes facilitated the transmission of antimicrobial genes by conjugation with *E. coli* J53. Our research shows that the transmission and adaptation of *Klebsiella pneumoniae* producing ESBLs is occurring in zoo environments, suggesting that zoos may be becoming important potential reservoirs for clinically important drug-resistant genes. It is therefore necessary to monitor the emergence and spread of drug-resistant gene strains in captive wild animals held in zoo environments.

## Introduction

The emergence and dissemination of antimicrobial resistance (AMR) in the environment has become a global concern. AMR has become an area of focus over the past two decades and is now recognized as a potential and serious threat to global public health (Tacconelli et al., 2018). AMRs can be disseminated rapidly through various pathways, including foodborne pathogens, insects, wastewater, pet, food-production, or wild animals (Yang Q.E. et al., 2019). There were more than 25 instances of human infectious disease outbreak reported during the period between 1990 and 2000, 11 of which were related to animals in farms, petting zoos, and zoos (Bender and Shulman, 2004). A human may be infected through direct or indirect contact with wild animals during interactive activities in zoos, a situation that is lesser discussed in relation to public health, and there has to date, been little research on the role of wild animals in zoos and the epidemiology of multidrug resistance (MDR). Some previous studies have shown that bacterial isolates from wild animals that live in close proximity to humans have stronger drug resistance compared to wild animals living in remote areas (Rolland et al., 1985; Cole et al., 2005; Skurnik et al., 2006; Kozak et al., 2009; Allen et al., 2011). Captive wild animals in zoos have more close contact with humans and, therefore, are potential natural reservoirs for AMRs and antibiotic-resistant bacteria.

Bacteria can meet the evolutionary challenge of combating antimicrobial chemotherapy by acquiring preexisting resistance determinants from the bacterial gene pool. This is achieved through the concerted activities of mobile genetic elements that can move within or between DNA molecules, which include insertion sequences, transposons, and gene cassettes/integrons, and those that are able to transfer between bacterial cells, such as plasmids and integrative conjugative elements. MDR bacteria from captive wild animals can carry various mobile genetic elements, for example, a study on a Czech zoo found that IncI1 harbored *bla*_CTX–M–1_ and *qnrS1* in *E. coli* (Dobiasova et al., 2013), integron (class I and II). Another study found plasmid carrying *bla*_CMY–26_,*qnr* and *aac(6′)-Ib-cr* in Gram-negative bacterial isolates from a Japanese zoo (Ahmed et al., 2007), and MDR *Salmonella Enterobacter* has been recovered from captive wild animals in Ohio (Farias et al., 2015). These studies indicate that these environments facilitate the emergence and dissemination of multidrug resistant pathogenic bacteria, posing a serious public health risk due to the interactions between humans and animals in these environments.

To further understand the routes of dissemination of AMRs harboring bacteria in a zoo, this study collected fresh animal feces samples as part of the routine monitoring of bacterial diseases in Zhengzhou zoo, Henan province, China. The *Enterobacteriaceae* in these samples were isolated, and we discovered an MDR *Klebsiella pneumoniae* isolate from a Red Kangaroo had severe drug resistance, including second-generation cephalosporins (Cefuroxime Sodium), third-generation cephalosporins (Ceftriaxone) and even, fourth-generation cephalosporins (Cefepime). A whole genome sequencing (WGS) analysis was then conducted to evaluate the relationship between the plasmid, drug-resistant gene related elements, and human clinical isolates.

## Materials and Methods

### Bacterial Isolates

*Enterobacteriaceae* was isolated from fresh fecal samples of animals at Zhengzhou Zoo, Henan province, China. Briefly, fecal samples were collocated into an Eppendorf tube with 500 μL sterile saline, which was gently shaken, and allowed to stand for 10 min. The supernatant was used to inoculate onto the MacConkey Agar (Beijing SanYao Science & Technology Development Co, Beijing, China) plate at 35°C for 18 h. The colonies with different morphologies and colors were then stored and subjected to further analysis. Species identification was carried out using a 16S rRNA sequence (TIANYI HUIYUAN, China).

### Drug Susceptibility Testing

Drug susceptibility testing was performed by the broth microdilution method (microbial susceptibility kit, BIO-KONT, China), according to the CLSI guidelines (CLSI, 2019). Fifteen antimicrobial drugs were used to screen MDR, including Ampicillin, Ampicillin/Sulbactam, Piperacillin/Tazobactam, Aztreonam, Cefuroxime sodium, Ceftriaxone, Cefepime, Ciprofloxacin, Levofloxacin, Meropenem, Colistin, Chloramphenicol, Trimethoprim/Sulfamethoxazole, Nitrofurantoin, and Amikacin. We tested drug susceptibility to Tetracycline and Doxycycline by the Kirby-Bauer disk diffusion method (OXOID, United Kingdom), and *E. coli* ATCC25922 was used as a quality-control strain.

### Whole-Genome Sequencing and Bioinformatics Analysis

Based on drug susceptibility testing, an MDR *Klebsiella pneumoniae* isolate (named M297-1) was identified in a Red Kangaroo. This multidrug resistant (MDR) bacteria was subjected to WGS using the Oxford Nanopore Technologies (ONT) MinION platform (Biomarker Technologies, China) (Ashton et al., 2015; Loman et al., 2015). Sequencing was then carried out according to the standard protocol provided by ONT, and high-quality genomic DNA was extracted by NanoDrop, Qubit, and 0.35% agarose gel electrophoresis for purity, concentration, and integrity. Large fragments of DNA were recovered by the BluePippin automatic nucleic acid recovery system. The library was constructed by ligation sequencing kit (SQK-LSK109 Ligation Sequencing Kit, Oxford Nanopore Technologies, United Kingdom), and DNA damage repair and terminal repair magnetic bead purification were used to connect and re-purify, and the Qubit library was quantified and sequenced on the machine.

A phylogenetic tree was constructed based on 16s rRNAs and *Klebsiella pneumoniae* isolate multilocus sequence typing (MLST) was conducted by using MLST 1.8 or PubMLST^1^. Plasmid replicon typing, plasmid multilocus sequence typing, and identification of resistance genes were performed using Plasmid Finder^2^ or pMLST 2.0, and Resfinder 2.0 or CARD, respectively. The comparison of the similarity between plasmids and known plasmids was conducted using PLSDB databases^3^. The plasmid sequence was annotated with DFAST^4^, and the genomic structure was compared in EasyFig. The comparative map of the plasmid genome was drawn by the Illustrator for Biological Sequences (IBS) software v1.0. (Liu et al., 2015) and modified manually. Transposons and insertion sequences were determined using ISfinder.

### Conjugation Experiments and the Evaluation of Plasmid Stability

To investigate the transferability of the plasmid in *Klebsiella pneumoniae* M297-l isolate, we performed conjugation assays with sodium-azide resistance *E. coli* J53 as a recipient strain. Briefly, overnight cultures of MDR *Klebsiella pneumoniae* M297-l as a donor and the recipient *E. coli* J53 strain were 1:10 mix and conducted on nitrocellulose membranes on a MacConkey Agar plate by incubation at 35°C for 16–20 h. After incubation, we subsequently diluted the 10-fold serial, mixed the culture in sterile saline, and aliquoted 100 μL of diluted culture onto MacConkey Agar plates supplemented with 20 mg/L of cefotaxime and 200 mg/L of sodium azide. *E. coli* transconjugants were screened by drug susceptibility testing. To further evaluate the stability of the plasmid of *E. coli* transconjugants, *E. coli* transconjugants were passaged continuously in MHB without antibiotics and detected in a McConkey Agar plate containing 20 mg/L cefotaxime according to previous reports (Walsh et al., 2011; Di Luca et al., 2017; Wein et al., 2019).

Similarly, we selected the conjugate strains based on the drug sensitivity test and used the Illumina sequencing platform (BGI, China) for high-throughput sequencing of the whole genome of the transconjugants plasmid.

#### Accession Numbers

The sequence data and details of the sequenced samples, including the date and location of the collection and source, were submitted to the GenBank. Accession numbers for Chr-M297-1, plasmid pM297-1.1, and pM297-1.2 from *Klebsiella pneumoniae* M297-1, respectively. *Klebsiella pneumoniae* M297-1 assembly contigs were deposited under study accession number CP051490, CP051491, and CP051492.

## Results

### MDR *Klebsiella pneumoniae* M297-1 Isolates From Red Kangaroo

Overall, 24 isolates were isolated from animal fecal samples in Zhengzhou Zoo. These included 33 isolates of *Escherichia coli* and 1 isolate of *Klebsiella pneumoniae*. The susceptibility profiles indicated that most of the isolates were susceptible to some of the common antimicrobial drugs in clinical use. The antimicrobial resistance of the isolates from the Red Kangaroo (*n* = 4) was serious (Table 1) as compared with other samples the isolate of *Klebsiella pneumoniae* (named as M297-1) had high resistance to major groups of antimicrobial drugs including group A (Ampicillin), group B (Cefuroxime, Cefepime, Ceftriaxone, Ciprofloxacin, Levofloxacin, Trimethoprim/Sulfamethoxazole, Ampicillin/Sulbactam), group C (Aztreonam, Chloramphenicol), and group U (Nitrofurantoin).

**TABLE 1:** The drug susceptibility profiles of *Enterobacteriaceae* isolated from Red Kangaroo.

Bacteria species	MIC for Piperacillin/Tazobactam, mg/L	MIC for Meropenem, mg/L	Non-susceptible phenotype^a^
*Escherichia coli*	<8/4	<0.25	N/A
*Escherichia coli*	<8/4	<0.25	SAM, F/M
*Escherichia coli*	<8/4	<0.25	AMP, FEP, C, SXT, F/M
*Klebsiella variicola*	<8/4	<0.25	N/A
*Citrobacter freundii*	<8/4	<0.25	AMP, ATM
*Morganella morganii*	<8/4	<0.25	AMP, ATM, CXM, CL, C
*Klebsiella pneumoniae*	<8/4	<0.25	AMP, SAM, ATM, CXM, CRO, FEP, C, CIP, LEV, SXT, F/M, TE, DO

*^*a*^AMP, Ampicillin; SAM, Ampicillin/Sulbactam; ATM, Aztreonam; CXM, Cefuroxime; CRO, Ceftriaxone; FEP, Cefepime; CL, Colistin; C, Chloramphenicol; CIP, Ciprofloxacin; LEV, Levofloxacin; SXT, Trimethoprim/Sulfamethoxazole; F/M, Nitrofurantoin; TE, Tetracycline; DO, Doxycycline. N/A, Not Applicable.*

### Genomic Structure of the Chr-M297-1 Harboring *bla*_DHA–3_, *bla*_SHV–1_, and *bla*_CTX–M–14_

The isolate was subjected to WGS analysis. The chromosomal DNA of M297-1 (named as Chr-M297-1) is 5,750,384 bp in size and exhibits 100% identity with a query coverage of 100% to the *K. pneumoniae* by Ribosomal Multilocus Sequence Typing (rMLST). Isolate M297-1 belongs to ST 290)^5^, has the highest homology with *Klebsiella pneumoniae* isolate (99.93%) from human clinical samples based on phylogenetic tree of 16s rRNA (Supplementary Figure S1). For ST290, only five isolates were recorded in the database, of which one strain was from a dairy cow and the other four strains were from human clinical samples. Chr-M297-1 possesses 9 gene islands and 1 prophage, 169 antimicrobial genes most importantly carrying 3 β-lactamase genes (*bla*_DHA–3_, *bla*_SHV–1_, and *bla*_CTX–M–14_), *fosA5*, *dfrA3*, *sul3*, *etc.* (Table 2). In addition, the resistance pump was mainly composed of ABC family genes, as well as MFS, SMR, and MATE family genes.

**TABLE 2:** Genomic information of *Klebsiella pneumoniae* isolate M297-1 from Red Kangaroo in Zhengzhou zoo, Henan province.

		PlasmidFinder	Number of gene islands	Number of prophage^a^	Movable resistance determinants^b^
Chromosome	Chr-M297-1		9	1	*sul3*, ***bla*_DHA–3_**, ***bla*_SHV–1_**, ***bla*_CTX–M–14_**
Plasmids	pM297-1.1	IncFIB(K)	5	N/A	*aph(3″)-Ib, aph(6)-Id*, *qnrS1*, ***bla*_CTX–M–14_**, ***bla*_TEM–191_**
	pM297-1.2	IncFII(K)	7	1	*sul1*(2), *sul2*, *aph(3″)*, *aph(6)-I*, *aph(3′)-Ia*, *aac(3)-IIa*, *aadA16*, *dfrA27*, *arr-3*, *aac(6′)-Ib-cr*, *qacEΔ1*, *floR*, *qnrB2*, *qnrS1*, *tetG*, *tetR*(2), ***bla*_TEM–1_**, ***bla*_CTX–M–3_**

*^*a*^N/A, No Prophage. ^*b*^*β*-lactamase genes are shown in bold. The number in parentheses indicates the number of drug resistance genes.*

### Genomic Structure of Plasmid Carrying Important Antimicrobial Genes

**FIGURE 1:**
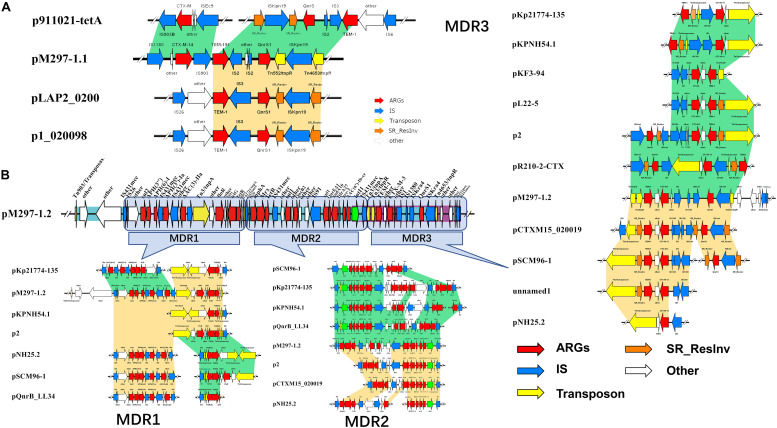
Antimicrobial genes, insertion sequences, and composite transposons in plasmids from MDR *K. pneumoniae* isolate M297-1. **(A)** pM297-1.1 and its similar linear property particle characteristics and comparison; **(B)** pM297-1.2 and its similar linear property particle characteristics and comparison. Dark green and golden shadows represent shared areas with high similarity (>90%), showing the relative positions of genes identified in the complete nucleotide sequences of these bacteria. These genes are marked with arrows, which represent the coding sequence and the associated transcription direction, and the size of the arrow is proportional to the length of the gene. The red shadow represents the antibiotic resistance gene, the blue shadow indicates the insertion sequence, the yellow shadow indicates the transposase gene, the green shadow indicates the integrase gene, and the orange shadow indicates the serine recombinase family protein gene. the blank indicates that it has nothing to do with drug resistance or unknown functional protein genes. Turquoise shadow, gene island; purple shadow, prophage.

*K. pneumoniae* isolate M297-1 contains two plasmids pM297-1.1 (222,864 bp) and pM297-1.2 (225,763 bp), which belong to IncFIB(K) and IncFII(K), respectively. pM297-1.1 contains five Genomic_islands, two β-lactamase genes (*bla*_CTX–M–14_ and *bla*_TEM–191_), two aminoglycoside resistant genes [*aph(3″)-Ib* and *aph(6)-Id*], quinolone resistance gene *qnrS1* and others. According to PLSDB database, only three plasmids were similar to pM297-1.1 focusing on the multidrug resistant region, which is *tnpA*-other-***bla*_CTX–M–14_**-*tnpA/*Tn903-***bla*_TEM–191_**—*tnpA/*IS2-other-***qnrS1***-*tnpR/Tn552*-ISKpn19-*tnpR/*Tn4653 (Figure 1). More specifically, there were conjugal transfer protein genes (*traABCDEGHIKLMNPQTUVX-trbBCEFI-finO*), Lactose permease, and two aminoglycoside resistant genes [transposase-*aph(3″)-Ib* and *aph(6)-Id*] co-located in the same larger Genomic_island 11 (42,044–101,359 bp).

pM297-1.2 contains 7 Genomic_islands and 1 prophage. Comparing to pM297-1.1, pM297-1.2 harbors more antimicrobial genes, including 2 extended-spectrum β-lactamase (ESBLs) genes (*bla*_CTX–M–3_ and *bla*_TEM–1_), 5 aminoglycoside resistance genes [*aph(6)-I,aph(3′)-Ia, aph(3)-IIa,aadA16, aph(6′)-Ib-cr*], fluoroquinolones resistant genes (*qnrB2* and *qnrS1*), *mphA, sul1, qacEΔ1* multidrug exporter, etc. Notably, pM297-1.2 carries chloramphenicol resistant genes (*floR*), tetracycline resistant genes (*tetG*), sulfonamide resistance gene (*sul1*, *sul2*) (Table 2), but pM297-1.1 does not contain these genes.

The genomic region of pM297-1.1 and pM297-1.2 were divided into different Multidrug Resistance regions according to the composition of antimicrobial genes and related antimicrobial genes. The pM297-1.1 possessed one multidrug resistance gene region (*tnpA*-other-*bla*_CTX–M–14_-*insC/*Tn903-*bla*_TEM–191_-*insD*-other-*insC21*-*qnrS1*-*tnpR/*Tn552-other-*tnpR/*Tn4653), which was entirely conserved in p911021-tetA, p1_020098, pLAP2_020009, NZ_CP040176.1 (similarity, >99.86%). This genomic region was flanked by *insC*/Tn903 and *insC21* sequences and co-harbored other genes, encoding ESBLs (*bla*_CTX–M–14_ and *bla*_TEM–191_, Figure 1A, pM297-1.1), and one gene conferring resistance to fluoroquinolones (*qnrS1*, Figure 1A, pM297-1.1).

The multidrug resistance region of pM297-1.2 appeared to consist of three parts, which are named MDR1, MDR2, and MDR3. MDR1 was bracketed by derivatives of Tn903, Tn3, and Tn1721(Tn21 subfamily) and harbored resistance genes against aminoglycoside, sulfonamide, and chloramphenicol. MDR2 carried one class I integron (intl1) and co-harbored other genes that have resistance to aminoglycoside, sulfonamide, rifampicin, and fluoroquinolones. Notably, MDR3 was bracketed by derivatives of Tn3 harboring two β-lactamases genes *bla*_CTX–M–3_ and *bla*_TEM–1_ and Tn4653 gene carrying one gene resistance to fluoroquinolones. Some plasmid, including pKp21774-135, pKPNH54.1, pKF3-94, pL22-5, p2, pR210-2-CTX, pCTXM15_020019, pSCM96-1, pNH25.2, were found to possess the MDR3 by a database search. These two plasmids evolved from other plasmids by inserting, deleting, or replacing.

### Traceability Analysis of These Two Plasmids

In the PLSDB database, five plasmids from *Klebsiella pneumoniae* in human clinical samples had high similarity to plasmid pM297-1.1. pM297-1.2 had high similarity to thirteen plasmids, of which 9 were derived from *Klebsiella pneumoniae*, and 1 from *Klebsiella variicola*, *Klebsiella quasipneumoniae*, *Klebsiella* sp., and *Escherichia coli* in human clinical samples from different regions (Supplementary Table S1).

### Conjugative Transfer and Stability of Plasmids Harboring MDR Regions

Transconjugants were obtained from mating experiments with *Klebsiella pneumoniae* M297-1 donors, in *E. coli* J53 recipients, at rates of 10^–5^ to 10^–6^ transconjugants per recipient cell. All of the 13 putative transconjugants that were tested were found to be the recipient background species and to be β-lactamase positive by antimicrobial susceptibility testing. Notably, 8 of the 13 transconjugants were resistant to sulfamethoxazole, chloramphenicol, and tetracycline, which was suggested to be transconjugants carrying with pM297-1.2 (Table 3). Another 4 strains were not resistant to the sulfamethoxazole, chloramphenicol, and tetracycline (Table 3), which was considered to be transconjugants carried pM297-1.1. All positive transconjugant colonies tested, resistance to cefotaxime, and remained detectable throughout the 6-day passage experiment, even in the absence of antibiotic selection.

**TABLE 3:** Drug susceptibility testing of *E. coli* transconjugants from *Klebsiella pneumoniae* M297-1 as donor and *E. coli* J53 as a recipient strain.

Agent^a^	J53-1	J53-2	J53-3	J53-4	J53-5	J53-6	J53-7	J53-8	J53-9	J53-10	J53-11	J53-12	J53-13
Ampicillin	R	R	R	R	R	R	R	R	R	R	R	R	R
Ampicillin/Sulbactam	S	S	S	I	I	I	S	I	I	I	S	R	R
Piperacillin/Tazobactam	S	S	S	S	S	S	S	S	S	S	S	I	S
Aztreonam	S	S	I	I	R	R	I	R	R	S	S	R	R
Cefazolin	R	R	R	R	R	R	R	R	R	R	R	R	R
Cefotaxime	R	R	R	R	R	R	R	R	R	R	R	R	R
Ciprofloxacin	S	R	I	I	R	R	I	R	R	R	R	R	R
Meropenem	S	S	S	S	S	S	S	S	S	S	S	I	S
Chloramphenicol	**S**	**S**	**R**	**R**	**R**	**R**	**S**	**R**	**R**	**R**	**S**	**R**	**R**
Trimethoprim/Sulfamethoxazole	**S**	**S**	**R**	**R**	**R**	**R**	**S**	**R**	**R**	**S**	**S**	**R**	**R**
Tetracycline	**S**	**S**	**R**	**R**	**R**	**R**	**S**	**R**	**R**	**R**	**S**	**R**	**R**
Doxycycline	**S**	**S**	**R**	**R**	**R**	**R**	**S**	**R**	**R**	**R**	**S**	**R**	**R**

*^*a*^Chloramphenicol, Trimethoprim/Sulfamethoxazole, Tetracycline and Doxycycline are shown in bold. S, susceptible; I, intermediate; R, resistance.*

We sequenced the whole genome of the J53-2 transconjugants plasmid, which is completely sensitive to Chloramphenicol, Trimethoprim/Sulfamethoxazole, Tetracycline, and Doxycycline, and also sequenced the transconjugants plasmid J53-12, which is highly resistant to Chloramphenicol, Trimethoprim/Sulfamethoxazole, Tetracycline, and Doxycycline. The results show that there was a complete genome of plasmid pM297-1.1 in J53-2 and a complete genome of pM297-1.2 in J53-12. This result confirmed that the two plasmids that were carried by strain M297-1 could be transferred to *E. coli*.

We confirmed that the two plasmids carried by *Klebsiella pneumoniae* M297-1 had strong transferability and stability in new host bacteria.

## Discussion

In this study, we reported firstly that the multidrug-resistant *Klebsiella pneumoniae* M297-1 was carried by healthy Red Kangaroo, which suggests that an asymptomatic animal host, which is likely to be ignored, may become an important reservoir for drug-resistant pathogens, such as *Salmonella entericus* (Perron et al., 2008). *Salmonella* isolated from captive wild animals in Ibadan, western Nigeria, has been observed to be resistant to sulfadiazine and penicillin (Falade and Durojaiye, 1976). Among the 232 isolates of Gram-negative bacteria isolated from mammals, reptiles, and birds raised in an Asakusa Zoo, Hiroshima Prefecture, Japan, 21.1% of Gram-negative bacteria carry at least one drug-resistant gene and have multiple drug-resistant phenotypes (Ahmed et al., 2007). The ESBLs and *fluoroquinolone* resistance genes detected in Czech zoos were associated with the transmission of specific *E. coli* clones and plasmids of specific incompatible groups between different animal species (Dobiasova et al., 2013). *S. aureus* was taken from zoo and wildlife in Germany from 2008 to 2016, and two isolates from juvenile red squirrels showed multiple drug resistant phenotypes (Fessler et al., 2018). The frequency of AMRs detected in animals living in human settlements is significantly higher than that in animals living in the wild (Grall et al., 2015; Kock et al., 2018). The isolates from wild animals show a similar pattern of drug resistance to *E. coli* from human clinical sources in their study areas (Jobbins and Alexander, 2015). Other studies have indicated that drug-resistant Salmonella isolates identified from zoo environments, including those from animals and their keepers, have been confirmed to be clone-related (Farias et al., 2015; Milton et al., 2018). Therefore, wild animals may be important hosts and storage hosts for the spread of drug-resistant bacteria, and human activities significantly affect the microbial community of captive wild animals in zoo environments, which are not necessarily monitored for such risks.

**FIGURE 2:**
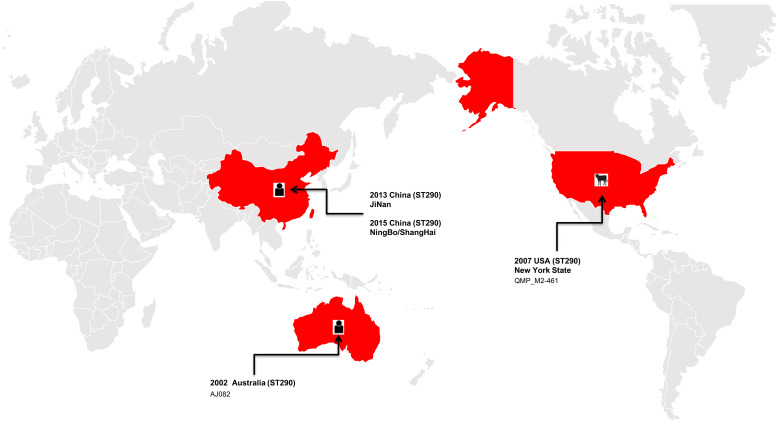
The distribution of the ST290 of *Klebsiella pneumoniae* isolates in the world, among which three isolates were found in China, one in the United States, and one in Australia.

In the present study, the ST290 sequence types *K. pneumoniae* M297-1 from a Red Kangaroo were closely related to that found in human clinical isolates, but only 5 cases of this sequence type have been reported worldwide, in the United States, Australia, and China (Figure 2). The plasmid of isolates carrying *bla*_KPC–2_, *bla*_IMP–8_, *bla*_TEM–1_, and *bla*_CTX–M–15_ gene transmission have been found in neonatal infections (Jin et al., 2017; Kong et al., 2020). The first case of nosocomial epidemic infection caused by NDM-5 metallo-β-lactamase ST290 isolates were reported in China (Wang et al., 2019). Therefore, our study further showed that zoo-derived MDR bacteria are closely related to human-derived MDR.

bGWAS provided comprehensive information about isolate M297-1. The whole genome analysis of M297-1 confirmed that its plasmids carried clinically related ESBLs genes including *bla*_TEM–1_, *bla*_TEM–191_, *bla*_CTX–M–3_, and *bla*_CTX–M–14_ (Table 2 and Figure 1). Since the late 1990s, the multidrug-resistant *Enterobacteriaceae* that produces ESBLs has become an important cause of urinary tracts and bloodstream infections in humans (Pitout and Laupland, 2008; Wyres et al., 2020). This is a rapidly evolving class of β-lactamases, usually from *bla*_TEM–1_, *bla*_TEM–2_, or *bla*_SHV–1_ genes, which can hydrolyze third-generation *cephalosporins* and *aztreonam* and can be inhibited by clavulanic acid (Paterson and Bonomo, 2005). Although new members of the ESBLs family are often found, the earlier *bla*_CTX–M–14_ and *bla*_CTX–M–15_ enzymes are prevalent in the world at large (Bush and Fisher, 2011; Bush and Bradford, 2020), while *bla*_CTX–M–3_ enzymes are prevalent mainly in Europe (Canton et al., 2008). Resistance to extended-spectrum cephalosporins such as ceftazidime, cefotaxime, and cefepime were often observed when ESBLs appeared in *Klebsiella* sp. (Babic et al., 2006). ESBLs were also detected on the M297-1 chromosome, but it also stably carried the ESBLs positive plasmids pM297-1.1 and pM297-1.2, indicating that drug resistance itself is not the only selection criterion for maintaining ESBLs cod plasmids. Other drug resistant genes, such as those of sulfonamides, fluoroquinolones, aminoglycosides, and tetracyclines and found on chromosomes and plasmids, suggest that the findings of this study may have evolved under the pressure of many antibiotics.

Mobile genetic elements, such as insertion sequence, transposon, integron, and prophage, can mobilize antibiotic resistance genes. The detection of *bla*_TEM_ and *bla*_CTX–M_ type β-lactamases genes in a variety of genetic backgrounds suggests that their mobilization may involve multiple mechanisms. In this study, for plasmid pM297-1.1, IS1380 is located upstream of *bla*_CTX–M–14_, and downstream we detected IS903, which has also located upstream of *bla*_TEM–191_ and *bla*_TEM–191_. This may also be related to its downstream truncated IS2 gene, and similar structures also exist in similar plasmids retrieved (p911021-tetA, MG288679.1; p1_020098, NZ_CP036307.1; pLAP2_020009, CP038004.1) (Figure 1). This seems to imply that the β-lactamase gene carried by pM297-1.1 may form a tandem structure of the drug resistance gene after multiple homologous recombinations. For the MDR 3 region of plasmid pM297-1.2, the resistance gene box of β-lactamase genes in Tn3-like transposon was *tnpA-tnpR-bla*_TEM–1_*-other-bla*_CTX–M–3_, and also located on the prophage. We found that a similar plasmid (pKF3-94, pL22-5, p2, pCTXM15_ 020019, pSCM96-1) also had a complete or truncated similar structure (Figure 1). There are usually two IS431mec insertion sequence genes from *Staphylococcus aureus* upstream and downstream of aminoglycosides, fluoroquinolones, sulfonamides, and tetracyclines in the pM297-1.2 MDR region. The structure of the intI1-gene cassette carried by the plasmid pM297-1.2 supports the concept of mobile elements to transfer antimicrobial genes between different bacteria. The drug resistance gene cassette [*aac(6′)-1b-cr-arr-3-dfrA27-aadA16]* is derived from the plasmid (ACC_NUCCORE : EU675686) carried by the multi-drug resistance *E. coil* isolated from the urine of patients in Huashan Hospital (Wei et al., 2009). This structure is completely preserved in pM297-1.2 from *Klebsiella pneumoniae* M297-1 (Figure 1) and is located on the Genomic Island. The drug resistance gene cassette mainly encodes β-lactamases, acetyltransferases, and nucleoside transferases, which do not require significant cell interaction and can be integrated into the metabolic network. Therefore, the interference from the existing genome is minimal, and it is the best example of a single gene corresponding to a single phenotype (Ghaly et al., 2020). In any case, this genetic factor will help bacteria evolve multiple determinants of antibiotic resistance in different habitats (Edge and Hill, 2005).

Asia is one of the centers of antibiotic resistance, and there are a number of drug-resistant strains including *K. pneumoniae*, and a large number of acquired gram-negative MDR strains have been found (Jean and Hsueh, 2011). The prevalent STs in Asia include ST15, ST23, ST14, and ST231. Among them, ST15 is very common, ST23 is significantly related to Southeast Asia, while ST14 and ST231 are significantly related to South Asia (Wyres et al., 2020). In this study, most of the plasmids retrieved from the PLSDB database were carried by *K. pneumoniae*. Among the *K. pneumoniae* isolates carrying highly similar plasmids to pM297-1.2, only two isolates of ST15 were isolated from Thailand and China, and one strain of ST23 and one strain of ST14 from China. The isolates carrying highly similar plasmids to pM297-1.1 did not detect the above four sequence types (Supplementary Table S1). As a result, the two plasmids in our study belong to IncF, a narrow host spectrum plasmid widely in *Enterobacteriaceae*, and can carry a variety of AMR genes and play a major role in the spread of specific antimicrobial genes (Carattoli, 2011; Rozwandowicz et al., 2018). Some studies showed that IncFIB and IncFII plasmids are effective vectors of β-lactamases. These plasmids can promote the transfer of antimicrobial genes when exposed to antibiotics (Rooney et al., 2019). *bla*_NDM_, *bla*_OXA_, and other genes spread in the *Enterobacteriaceae* flora of medical facilities by relying on these plasmids (Simner et al., 2018; Wu et al., 2019; Strydom et al., 2020), while *bla*_CTX–M_ can become the dominant gene carried by IncFII plasmids in France and China (Du et al., 2012; Dahmen et al., 2013). It seems that *bla*_CMY–42_ is replacing *bla*_CTX–M–15_ in some areas (Paul et al., 2020). In particular, some IncFIB cannot only express a high level of drug resistance but also enhance the virulence of *K. pneumoniae* after conjugation with *K. pneumoniae* (Yang X. et al., 2019). The results of this suggest that the co-prevalence of plasmid IncFIB and IncFII in the same isolates may lead to serious public health problems.

**FIGURE 3:**
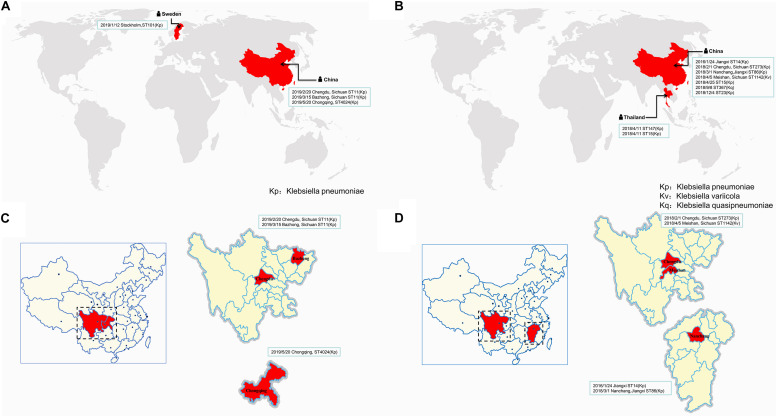
The distribution of different isolates carrying similar plasmids in the world. The distribution of different isolates carrying similar plasmids that are highly similar to plasmids pM297-1.1 **(A)** and pM297-1.2 **(B)**, while **(C,D)** indicate the distribution in China. The reporting date, region, and ST typing of the isolates are shown in the box.

**FIGURE 4:**
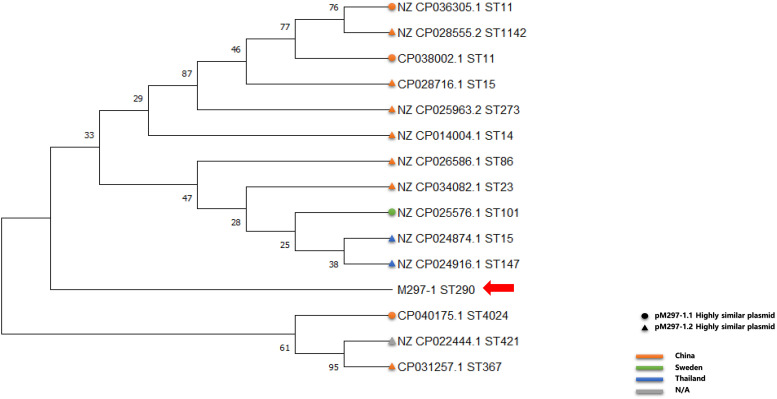
Phylogenetic relationship between reference *K. pneumoniae* isolates carrying similar plasmids and M297-1 based on 16s rRNA sequences. The circle indicates the strain carrying highly similar plasmids to pM297-1.1, the triangle indicates the strain carrying highly similar plasmids to pM297-1.2, and the red arrow points to M297-l. Orange, China; light green, Sweden; dark blue, Thailand; gray, missing information.

Similar plasmids were found by Sweden, Thailand, and China, respectively. Except for one isolate in Sweden and two isolates in Thailand, the other isolates carrying similar plasmids are mainly distributed among the southern cities of Sichuan province, China (Figure 3 and Supplementary Table S1). It has been suggested that during the period from 2010 to 2019, the epidemic distribution of this drug-resistant plasmid was gradually spreading in China, and the difference in carrying drug-resistant genes indicates that it is evolving. However, the phylogenetic analysis of the related isolates showed that M297-1 is located on a different branch with other *K. pneumoniae* isolates carrying similar plasmids (Figure 4). Since strain M297-1 comes from red kangaroo samples, while other strains carrying similar plasmids come from human clinical samples, it is speculated that the reason why M297-1 and other *K. pneumoniae* carrying similar plasmids are in different branches may be closely related to their hosts.

## Conclusion

In conclusion, this study demonstrated the high resistance of 13 drugs including *Ceftriaxone* and *Cefepime* on MDR *K. pneumoniae* isolate, taken from healthy Red Kangaroos in Zhengzhou zoo, China. This is the first report on a *K. pneumoniae* M297-1 (ST290) from a wild animal carrying *bla*_DHA–3_, *bla*_SHV–1_, *bla*_CTX–M–14_, *bla*_TEM–191_, *bla*_TEM–1_, and *bla*_CTX–M–3_ in China and two conjugal transferable plasmids that co-harbor other antimicrobial genes: *aph(3′)-Ia, aph(3″)-Ib, aph(6)-Id, aac(3)-IIa, aac(6′)-Ib-cr, aadA16, qnrB2, qnrS1, qacEΔ1, mphA, sul1*, and *dfrA27*. This research confirmed that there is a close relationship between drug-resistant strains carried by wild animals in zoos and human clinical isolates. This suggests that zoos may be becoming important reservoirs for clinically important MDR isolates, which pose a serious potential public health risk. These potential reservoirs should not be ignored and monitoring these environments is of vital importance in preventing major threats to public health in the future.

## Data Availability Statement

The sequence data and details of the sequenced samples, including the date and location of collection and source, were submitted to the GenBank. Accession numbers for Chr-M297-1, plasmid pM297-1.1, and pM297-1.2 from Klebsiella pneumonia M297-1, respectively. Bioproject IDs PRJNA624988 and PRJNA624988, respectively. Klebsiella pneumoniae M297-1 assembly contigs are deposited under study accession number CP051490, CP051491, and CP051492, BioProject ID PRJNA624988.

## Ethics Statement

The animal study was reviewed and approved by the Animal Experimental Ethics Committee of Institute of Zoology (No. GIABR20191104).

## Author Contributions

CW and JQ designed the study. XW, QK, JZ, ZL, and FJ isolated and identified isolates. JL, JY, CZ, TJ, GD, and SL collected samples and analyzed the data. XW, CW, and JQ wrote and edited the manuscript. All authors contributed to the article and approved the submitted version.

## Conflict of Interest

The authors declare that the research was conducted in the absence of any commercial or financial relationships that could be construed as a potential conflict of interest.

## Footnotes

1 http://pubmlst.org/

2 https://cge.cbs.dtu.dk/services/PlasmidFinder/

3 https://ccb-microbe.cs.uni-saarland.de/plsdb/

4 https://dfast.nig.ac.jp/

5 https://pubmlst.org/rmlst/
