# Supplementary material for: Characteristics and Epidemiology of Extended-Spectrum β-Lactamase-Producing Multidrug-Resistant Klebsiella pneumoniae From Red Kangaroo, China
Source: Front Microbiol. 2020 Oct 14;11:560474. doi: 10.3389/fmicb.2020.560474 (PMC7591395; doi:10.3389/fmicb.2020.560474)

Supplementary Figure S1. Phylogenetic relationship between Klebsiella pneumoniae isolate M297-1 and reference strain from human clinical samples based on the 16s rRNA.

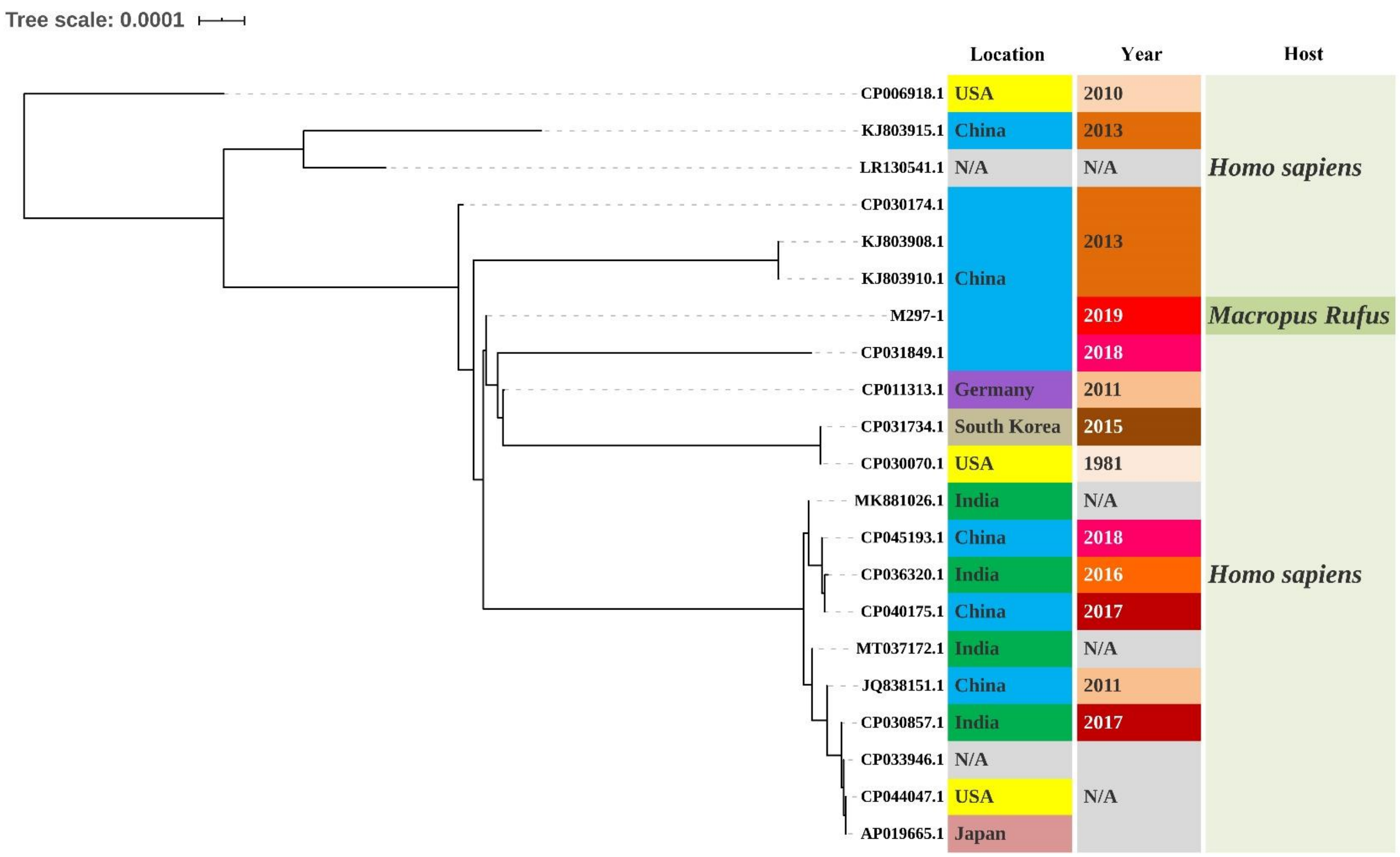

Supplement: Supplementary file 1 [file Presentation_1.pdf]
