# Supplementary material for: Characteristics and Epidemiology of Extended-Spectrum β-Lactamase-Producing Multidrug-Resistant Klebsiella pneumoniae From Red Kangaroo, China
Source: Front Microbiol. 2020 Oct 14;11:560474. doi: 10.3389/fmicb.2020.560474 (PMC7591395; doi:10.3389/fmicb.2020.560474)
Supplement: Supplementary file 2 [file Table_1.docx]

**Supplementary Table S1.Comparison between reference plasmid and pM297-1.1 and pM297-1.2 from *Klebsiella pneumoniae* M297-1 from Red Kangaroo in Zhengzhou zoo, Henan province.**

| Similar plasmid† | ACC_NUCCORE | Plasmid name | CreateDate | Location | Host | Sputum sample | PlasmidFinder | Taxon name | MLST |
| --- | --- | --- | --- | --- | --- | --- | --- | --- | --- |
| pM297-1.1 | MG288679.1 | p911021-tetA | 2018/4/16 | N/A | N/A | N/A | IncFIB(K) | *K.pneumoniae* | N/A |
|  | NZ_CP025577.1 | p08EU827_1 | 2019/1/12 | Sweden:Stockholm | *Homo sapiens* | Bacterial isolate | IncFIB(K) | *K.pneumoniae* | ST101 |
|  | NZ_CP036307.1 | p1_020098 | 2019/2/20 | China:Chengdu,Sichuan | *Homo sapiens* | Pure Culture | IncFIB(K) | *K.pneumoniae* | ST11 |
|  | CP038004.1 | pLAP2_020009 | 2019/3/15 | China:Bazhong,Sichuan | *Homo sapiens* | Pure Culture | IncFIB(K) | *K.pneumoniae* | ST11 |
|  | NZ_CP040176.1 | unnamed1 | 2019/5/20 | China:Chongqing | *Homo sapiens* | Whole organism | IncFIB(K) | *K.pneumoniae* | ST4024 |
| pM297-1.2 | NC_013950.1 | pKF3-94 | 2010/3/19 | N/A | N/A | N/A | IncFII(K) | *K.pneumoniae* | N/A |
|  | NZ_CP011334.1 | unnamed | 2015/5/6 | N/A | N/A | N/A | IncFII(K) | *E.coli* | ST290 |
|  | NZ_CP014005.1 | unnamed1 | 2016/1/24 | China:Jiangxi | *Homo sapiens* | N/A | IncFII(K) | *K.pneumoniae* | ST14 |
|  | NZ_CP025966.2 | pQnrB_LL34 | 2018/2/1 | China:Chengdu,Sichuan | *Homo sapiens* | Culture | IncFII(K) | *K.pneumoniae* | ST273 |
|  | NZ_CP026588.1 | p2 | 2018/3/1 | China:Nanchang,Jiangxi | *Homo sapiens* | Tissue sample | IncFII(K) | *K.pneumoniae* | ST86 |
|  | NZ_CP028553.2 | pCTXM15_020019 | 2018/4/5 | China:Meishan,Sichuan | *Homo sapiens* | Culture | IncFII(K) | *K.variicola* | ST1142 |
|  | NZ_CP024917.1 | pKPNH54.1 | 2018/4/11 | Thailand | *Homo sapiens* | N/A | IncFII(K) | *K.pneumoniae* | ST147 |
|  | NZ_CP024876.1 | pNH25.2 | 2018/4/11 | Thailand | *Homo sapiens* | N/A | IncFII(K) | *K.pneumoniae* | ST15 |
|  | NZ_CP028717.1 | pSCM96-1 | 2018/4/25 | China | N/A | Sputum sample | IncFII(K) | *K.pneumoniae* | ST15 |
|  | NZ_CP031262.1 | pL22-5 | 2018/9/8 | China | *Homo sapiens* | N/A | IncFII(K) | *K.quasipneumoniae* | ST367 |
|  | NZ_CP034085.1 | pR210-2-CTX | 2018/12/4 | China | *Homo sapiens* | Cell culture | IncFII(K) | *K.pneumoniae* | ST23 |
|  | NZ_CP022442.1 | unnamed1 | 2019/1/12 | N/A | N/A | N/A | IncFII(K) | *Klebsiella sp.* | ST421 |
|  | MG878868.1 | pKp21774-135 | 2019/2/28 | N/A | N/A | N/A | IncFII(K) | *K.pneumoniae* | N/A |
| †N/A,Unknown | | | | | | | | | |
